# Pulmonary epithelioid trophoblastic tumor without a primary uterine lesion: a case report

**DOI:** 10.3389/fmed.2026.1808463

**Published:** 2026-03-30

**Authors:** Chuntian Ma, Yuhao Liu, Weijun Wang, Wei Li

**Affiliations:** 1 Department of Medical Imaging, Baoji Central Hospital, Baoji, Shaanxi, China; 2 Department of Clinical Medicine, General Hospital of Ningxia Medical University, Yinchuan, Ningxia, China

**Keywords:** epithelioid trophoblastic tumor, fertility-sparing treatment, isolated pulmonary lesion, solitary nodules in the lung, video-assisted thoracoscopic surgery

## Abstract

Epithelioid trophoblastic tumor (ETT) is a rare malignant gestational trophoblastic neoplasm arising from the malignant transformation of chorionic-type intermediate trophoblastic cells, with important clinical implications, particularly regarding fertility-sparing management in selected patients. At present, most of the ETT cases reported in the literature are characterized by primary lesions of reproductive system origin, with or without distant metastasis. Here, we report a case of isolated pulmonary ETT without a primary uterine lesion. The patient underwent video-assisted thoracoscopic surgery (VATS) single-port right lower lobectomy. The postoperative pathology and immunohistochemistry confirmed pulmonary ETT. This article also reviews its clinical features, imaging manifestations, histopathological characteristics, and treatment options in the context of the literature, which helps to gain a deeper understanding of this rare isolated pulmonary ETT and provides new ideas for clinical diagnosis.

## Introduction

Epithelioid trophoblastic tumor (ETT) is a rare type of gestational trophoblastic neoplasia originating from chorionic-type intermediate trophoblast cells. It represents approximately 1.4–2.0% of all gestational trophoblastic diseases, with only a handful of cases reported in the literature (1). Histologically, ETT displays characteristics of both trophoblastic tumors and carcinoma, predominantly resembling carcinoma-like (epithelioid) features in most instances (2). Endometrial tissue tumors typically originate in the uterus, with the lungs serving as the principal site for distant metastasis (3). Diagnosing isolated pulmonary ETT can be challenging when there is no identifiable primary lesion in the reproductive system. This challenge arises from the absence of specific clinical symptoms and sensitive tumor markers (4, 5), making early detection difficult. Moreover, isolated pulmonary ETT is frequently misdiagnosed. It is often confused with other tumors or lung diseases, such as lung cancer, pulmonary tuberculosis, or pulmonary cysts, on standard X-rays and computed tomography (CT) scans. The misdiagnosis arises due to the rarity of pulmonary ETT, which can occasionally resemble other malignancies. Given the aggressive potential of ETT and its distinct therapeutic implications, early recognition is clinically crucial. This study performed a retrospective analysis of clinical data and CT imaging results from patients diagnosed with pulmonary ETT who lacked a primary uterine lesion as verified by postoperative pathology at our hospital. It seeks to improve radiologists’ comprehension of pulmonary ETT and diminish the occurrences of misdiagnosis and overlooked diagnoses.

### Case presentation

A 29-year-old married female patient was diagnosed with a space-occupying lesion in the lower lobe of the right lung during a physical examination and was subsequently admitted to the Department of Thoracic Surgery for further evaluation and management. The patient has not exhibited any clinical symptoms related to respiratory disease since the disease’s onset. The patient denied vaginal bleeding, abdominal pain, or abdominal distension. On pulmonary examination, breath sounds were coarse bilaterally. No dry or wet rales were detected. Gynecologic examination revealed a normal vagina and unremarkable bilateral adnexa. The cervix was hypertrophic with mild erosion. The patient has experienced 2 pregnancies, yielding 1 live birth and 1 miscarriage that transpired 4 years prior. She experienced amenorrhea for the past 2 months. Following treatment with traditional Chinese medicine, her menstrual cycle resumed, now occurring bimonthly and lasting 10 days each time.

In various laboratory tests, an elevated serum beta-human chorionic gonadotropin (*β*-hCG) level of 659.44 mIU/mL (normal range <5 mIU/mL) was detected. In women of reproductive age presenting with amenorrhea and elevated serum *β*-hCG, intrauterine or ectopic pregnancy must be prioritized in the differential diagnosis. Accordingly, a transvaginal ultrasound (TVUS) was performed, revealing an unremarkable uterus and bilateral adnexa with no evidence of a gestational sac or pregnancy.

A noninvasive evaluation was conducted using cervical liquid-based cytology. The results showed no evidence of intraepithelial lesions or malignant cells. The patient underwent hysteroscopy and endometrial biopsy. Histopathology revealed a proliferative phase endometrium, with no evidence of chorionic villi or trophoblastic components. Based on these findings, together with the clinical presentation and imaging results, no evidence of epithelioid trophoblastic tumor involving the reproductive system was identified.

**Figure 1:**
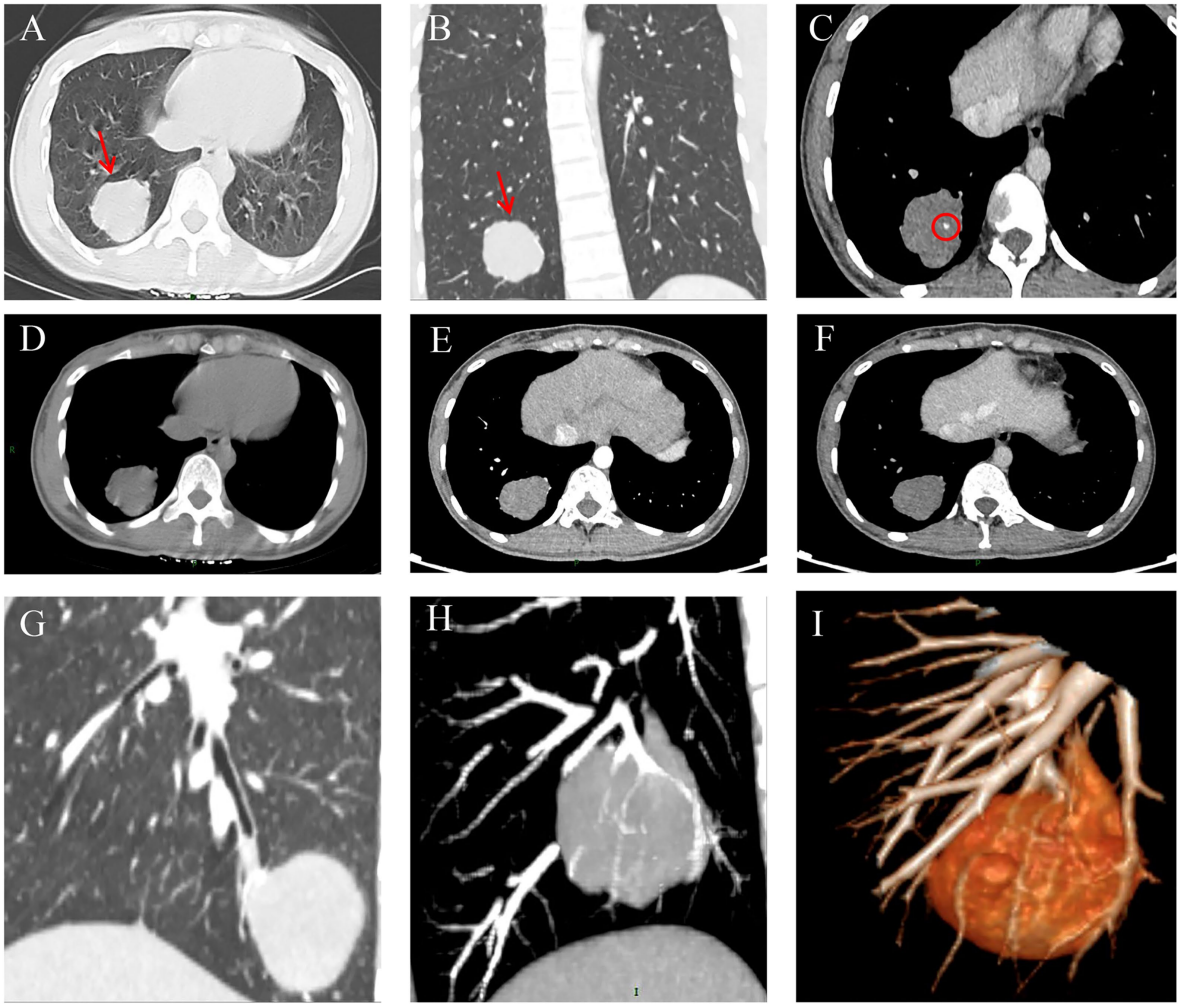
CT images of the patient. The patient’s chest CT shows a spherical soft tissue mass in the right lung’s lower lobe (**A,B**, red arrow). **C** and **D** show uneven density and calcific shadowing, as indicated by the red circle in **(C)**. Lesion enhancement on chest CT showed mild to moderate enhancement with a CT value of 45 HU during the arterial phase **(E)** and additional enhancement with a CT value of 56 HU during the venous phase **(F)**. No mediastinal lymph nodes were enlarged. MPR showed the lesion was related to the adjacent bronchus **(G)**. MIP **(H)** and VR **(I)** showed many vascular shadows around the lesion.

A CT scan (Figures 1A–I) showed a round soft tissue mass located in the lower lobe of the right lung, measuring about 4.2 × 3.5 × 2.6 cm. The mass exhibited well-defined margins and shallow lobulation, with a CT value of about 23 Hounsfield Unit (HU), inhomogeneous density, and calcified shadows within the lesion. The arterial phase showed mild-to-moderate enhancement, with a CT value of about 45 HU, and the venous phase showed further enhancement, with a CT value of about 56 HU. The lesion was surrounded by multiple vascular shadows and was connected to the neighboring bronchus. Bronchoscopy showed no evidence of current active bacterial, viral, or fungal infection in the lumen of the trachea. Lavage fluid examination of the lower lobe of the right lung revealed the presence of numerous ciliated columnar epithelial cells, histiocytes, neutrophils, lymphocytes, and a few squamous epithelial cells.

The patient presented with a richly vascularized lesion in the right lower lobe of the lung, which appeared to be lobulated. Multiple blood vessels were seen around the lesion connected to the adjacent bronchus. Given the possibility of a malignant lung tumor, the thoracic surgeon recommended a video-assisted thoracoscopic surgery (VATS) single-port wedge resection of the right lower lobe, taking into account the lesion’s location, the feasibility of complete resection, and the desire to minimize surgical trauma.

Following the induction of general anesthesia, one-lung ventilation (OLV) was established via a left-sided double-lumen endobronchial tube (DLT), with its position strictly verified by fiberoptic bronchoscopy after transitioning the patient to the left lateral decubitus position. Passive collapse of the right lung was achieved by opening the DLT to atmospheric pressure. The procedure was performed using a biportal video-assisted thoracoscopic surgery (VATS) approach, facilitated by a Karl Storz 4 K ultra-high-definition system (Karl Storz, Tuttlingen, Germany) and a 10-mm, 30-degree fiber-optic endoscope. A 1.0-cm camera port and a 4.0-cm utility incision were placed at the eighth intercostal space (ICS) on the mid-axillary line and the fifth ICS on the anterior axillary line, respectively. Intraoperative exploration identified a 4.0-cm firm mass in the right lower lobe, for which an initial wedge resection was performed using endoscopic linear staplers. Subsequent frozen section analysis indicated an epithelioid malignant tumor, necessitating a conversion to a formal right lower lobe lobectomy with systematic mediastinal lymphadenectomy. The right lower lobe vein, bronchus, and the main arterial stem were individually dissected and transected with endoscopic staplers. After ensuring adequate lung re-expansion and confirming the absence of air leaks under a saline seal, a 30-Fr chest tube was secured via the eighth ICS. No postoperative complications or adverse events occurred during the patient’s hospitalization. The postoperative course was uneventful, with satisfactory wound healing and no evidence of surgical, respiratory, or systemic complications.

**Figure 2:**
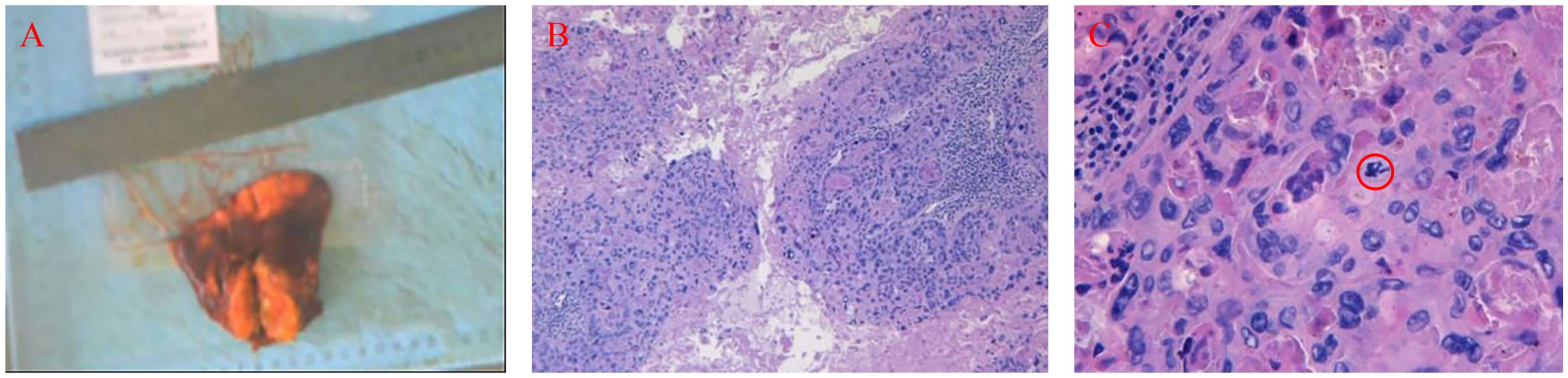
Histology of postoperative specimens. Excised specimen macroscopic features **(A)** The tubercle slice was distinguishable from the neighboring tissue with its earthy yellow color, homogenous texture, and localized necrosis. A 100-fold HE staining **(B)** showed that the tumor tissue was mostly solitary epithelioid cells organized in a nested lamellar configuration, near eosinophilic, fibrous, hyalinoid substances, and necroclasts, creating a “map” around the remaining tumor cells’ extensive necrosis. The 200-fold HE staining under the microscope **(C)** showed that tumor cell cytoplasm was abundant and pale pink or translucent. The nucleus is heteromorphic and divides (red circle).

The gross specimen after surgery (Figures 2A–C) showed that the tumor nodule was tan in color and homogeneous in texture, with areas resembling necrotic flesh. Additionally, it displayed clear boundaries with the surrounding tissue. Histological examination revealed that the tumor tissue was composed of relatively uniform epithelioid cells arranged in nests and sheets. These tissues were closely associated with eosinophilic, fibrous, and hyaline materials along with areas of necrotic debris. Extensive necrosis surrounding viable tumor cells formed a “map-like” pattern, and mitotic figures were observed. Immunohistochemical examination showed the following results: CK (cytokeratin) (+), Vimentin (−), S-100 (−), P63 (tumor protein p63) (+), P40 (focally weak+), CK5/6 (−), CK7 (+), TTF-1(thyroid transcription factor-1) (−), LCA (−), CR (−), Ki-67 (>50%), EMA (−), NapsinA (−), GATA-3 (GATA binding protein 3) (+), CK (LMW) (+), MUC-4 (−), inhibin-α (inhibin alpha) (focally+), HCG-a (focally+), P53 (patchy+), HPL (human placental lactogen) (−), and *β*-catenin (membranous+). No metastasis was found in the lymph nodes. The morphological and immunohistochemical findings, in conjunction with the patient’s clinical history, confirmed the diagnosis of ETT.

The patient was ultimately diagnosed with isolated pulmonary ETT. Due to the patient’s significant wish to maintain fertility, a hysterectomy was not conducted.

Aligned with the National Comprehensive Cancer Network (NCCN), International Federation of Gynecology and Obstetrics (FIGO), and European Society for Medical Oncology (ESMO) guidelines, a multidisciplinary team (MDT) comprising specialists in gynecology, thoracic surgery, and pathology formulated a management strategy centered on fertility-sparing surgery and rigorous long-term surveillance. Postoperatively, serum *β*-hCG levels were monitored weekly during the first month and showed a significant decline, remaining within the normal range (<1.20 mIU/mL) for four consecutive weeks. The monitoring frequency was subsequently adjusted to monthly. After three consecutive monthly negative results, the interval was extended to every 6 months. Throughout the 4-year follow-up period, *β*-hCG levels remained consistently normal. Regarding imaging, chest CT and TVUS were performed semi-annually for the first year with no evidence of recurrence or metastasis. From the second postoperative year to the present, annual imaging surveillance has remained negative. As no obvious recurrence was observed during the first year of follow-up, the patient declined postoperative chemotherapy.

The 29-year-old married patient with one live birth and a miscarriage 4 years prior prioritized fertility preservation throughout her clinical management and reported a satisfactory postoperative quality of life without psychological or physical distress. Her family medical history was unremarkable for gestational trophoblastic disease or related malignancies.

## Discussion

Gestational trophoblastic neoplasia comprises a distinct category of pregnancy-associated tumors, including invasive mole (IM), choriocarcinoma (CCA), placental site trophoblastic tumor (PSTT), and ETT. The pathogenesis of ETT remains insufficiently comprehended to date (6)^.^ ETT may arise subsequent to any pregnancy event, including full-term delivery, molar pregnancy, or spontaneous abortion, or as a consequence of a prior gestational trophoblastic neoplasm (7)^.^ The patient in this case had a history of miscarriage 4 years earlier, aligning with the literature reports.

ETT predominantly manifests in women of reproductive age, with a mean onset age of 36 years. Nonetheless, literature also documents instances occurring in postmenopausal women (8). Patients typically exhibit irregular vaginal bleeding; however, they may also experience abdominal pain, abdominal distension, amenorrhea, and symptoms associated with metastatic lesions. The patient exhibited an irregular menstrual cycle and amenorrhea. In contrast to patients with CCA, who often present with serum *β*-hCG levels exceeding 10,000 mIU/ml, patients with ETT typically show only modest elevations in β-hCG levels (<2,500 mIU/ml). The patient’s initial serum *β*-hCG level (659.44 mIU/ml), while elevated, was characteristically lower than levels typically associated with gestational CCA. Consistent with current literature, ETT and PSTT frequently present with only moderate β-hCG elevations. Postoperatively, a rapid decline to normal range (<1.20 mIU/ml) sustained over four consecutive weekly measurements within the first month confirmed biochemical remission following the complete resection of the pulmonary lesion. A distinct retrospective study indicates that 30% of ETT cases arise in the uterine corpus, 50% in the lower uterine segment and cervical canal, and 20% may manifest outside the uterus, including metastatic locations such as the lungs and intestines (9). Certain researchers have examined the metastatic locations of ETT and determined that the lungs represent the predominant site of metastasis, followed by the liver and brain.

A limited number of ETT cases have exhibited a distinctive clinical phenomenon, marked by isolated pulmonary ETT without a discernible primary source in the reproductive system. Two principal hypotheses have been put forth to elucidate the underlying pathogenesis of this disease (10). One hypothesis suggests that primary trophoblastic stem cells may spread to the lungs early in gestation, where they later undergo malignant transformation into ETT. An alternative hypothesis is that the isolated pulmonary lesion could be a metastasis from a regressed primary uterine lesion. In this case, the patient presented with recent amenorrhea and a history of abortion 4 years prior. A routine physical examination revealed a mass in the right lower lobe of the lung, but no primary lesion was identified in the reproductive system. Given the lack of a primary lesion, *β*-hCG monitoring was essential in guiding the diagnosis, suggesting a trophoblastic origin for the pulmonary mass. Postoperative pathology, along with the FIGO 2000 classification, confirmed the final diagnosis of ETT.

When patients present with isolated pulmonary ETT without a primary lesion in the reproductive system, preoperative diagnosis is extremely challenging. Based on case reports in the domestic and international literature (11–14). The characteristics of pulmonary ETT as observed in chest CT imaging can be summarized as follows: Pulmonary ETT typically presents as either solitary or multiple nodules or masses within the lung, with a solitary lesion occurring more frequently. When solitary, the right lung is more frequently involved, possibly due to its larger volume and richer blood supply. In cases of multiple lesions, the nodules or masses vary in size and are randomly distributed throughout both lungs. The margins of the lesions may be clear or indistinct, and some may appear lobulated. In some cases, necrosis or cystic changes may be observed within the lesions. On contrast-enhanced scans, the lesions usually show mild to moderate homogeneous or heterogeneous enhancement, with multiple vascular shadows visible around them. Calcification can occur in ETT and may manifest in various forms, such as punctate, patchy, or ring-like patterns. According to some reports (10), calcification can be a characteristic feature of ETT, as it is more common in epithelioid trophoblastic tumors compared to PSTT and CCA. In a small number of cases (14, 15), cavitary lung lesions may occur, characterized by irregular wall thickness. Perilesional ground-glass opacity with indistinct margins may be seen around pulmonary ETT lesions, suggesting local inflammation or hemorrhage. In some cases, the presence of pleural thickening and pleural effusion may complicate the diagnostic process. Pulmonary ETT is rare in patients, which can help distinguish it from more aggressive pulmonary malignancies. While pulmonary malignancies often present with rapid progression, significant mass effect, and more aggressive histopathologic features, ETT tends to have a more indolent course. This slower clinical progression, coupled with the absence of the typical features seen in more common pulmonary cancers, further supports the diagnosis of ETT in our case. Additionally, the sustained low-level *β*-hCG positivity and the patient’s clinical history, which did not suggest other malignancies, further reinforce the rare nature of pulmonary ETT and its distinction from other primary or metastatic lung tumors. In the present case, the patient presented with a solitary, well-demarcated, lobulated lesion in the right lower lobe, with calcification within the lesion and mild to moderate homogeneous enhancement observed on a contrast-enhanced scan. Consistent with the literature, no enlarged lymph nodes were observed in the mediastinum or hilar regions.

The final diagnosis of pulmonary ETT relies on pathological examination and immunohistochemical results (16). As demonstrated in Table 1, p63 serves as a critical diagnostic hallmark for ETT, exhibiting diffuse nuclear staining, which distinguishes it from PSTT and CCA. While ETT and PSTT share some morphological features, the strong expression of hPL is characteristic of PSTT, whereas ETT is more likely to express p63 and GATA-3. In the present case, the tumor exhibited p63 (+), GATA-3 (+), and focal hCG (+), alongside a Ki-67 index of >50%. Although the Ki-67 index in our patient was higher than the typical range reported in some literature, the overall IHC profile and ‘map-like’ necrosis pattern remained highly consistent with the diagnosis of ETT.

**Table 1:** Differential immunohistochemical profiles of ETT, PSTT, and CCA.

Marker	ETT	PSTT	CCA
p63	Diffuse and strong nuclear expression (+)	Negative or rare focal expression (−)	Negative (−)
hPL	Focal or negative (+/−)	Diffuse and strong positive (+)	Focal positive (+/−)
β-hCG	Focal or weak positive (+/−)	Focal or weak positive (+/−)	Diffuse and strong positive (+)
GATA-3	Positive (+)	Negative or focal positive (−/+)	Positive (+)
HLA-G	Positive (+)	Positive (+)	Positive (+)
Ki-67 Index	Usually 10–30% (moderate)	Usually 10–30% (Moderate)	Frequently >50% (High)
Inhibin-α	Focal positive (+/−)	Positive (+)	Focal positive (+/−)

ETT, Epithelioid Trophoblastic Tumor; PSTT, Placental Site Trophoblastic Tumor; CCA, Choriocarcinoma.

Surgical resection remains the primary therapeutic strategy for ETT, particularly in localized cases, given its inherent chemoresistance—a characteristic shared with PSTT. According to NCCN, FIGO, and ESMO guidelines, surgical management is stage-dependent. For Stage I disease (confined to the uterus), the standard approach involves total hysterectomy and salpingectomy; routine oophorectomy is generally discouraged, whereas lymphadenectomy is considered only for large or deeply invasive tumors. In Stage III cases with pulmonary involvement, hysterectomy with salpingectomy and excision of metastatic lesions is recommended when feasible (17). Notably, the prognosis for isolated pulmonary ETT mirrors that of Stage I disease, with no fatalities reported among the 10 documented cases (Table 2). In the previously reported 10 cases of isolated pulmonary ETT, operations were performed (Table 2). Among these cases, 7 cases underwent thoracic surgery without hysterectomy (T-H). In comparison, 3 cases had the isolated lung lesions removed along with total hysterectomy (T + H), where postoperative pathology revealed a benign disease in the uterus. Patients managed with isolated pulmonary resection without hysterectomy achieved complete remission and favorable prognoses without evidence of recurrence or metastasis. Huang et al. (11) reported that complete resection of all isolated pulmonary lesions during the initial operation, rather than additional hysterectomy, may be the key to reducing the risk of recurrence in isolated pulmonary ETT. Pulmonary lesion resection without hysterectomy is thus a feasible approach for patients with isolated pulmonary ETT who wish to preserve fertility, a conclusion supported by the findings of Liu et al. (13). Serial postoperative *β*-hCG monitoring alongside periodic chest CT and transvaginal ultrasonography remains essential to detect potential disease progression or recurrence in this patient cohort.

**Table 2:** Clinical features of patients with isolated pulmonary ETT in the literature.

Authors	Patient No.	Age, years	Symptoms	Preceding pregnancy	Provisional diagnosis	Diagnostic method	Number of metastases in lungs	Pretreatment hCG, mIU/mL	Surgical resection of pulmonary lesion	Hysterectomy	Preoperative chemotherapy	Postoperative chemotherapy	Initial remission, months	Recurrence	Subsequent treatment	Follow-up, survival, months
Huang et al. (11)	1	32	Amenorrhea and chest pain	term pregnancy	Invasive cancer	VATS	2	52.0–75.3	Wedge resection of both lungs	−	MTX	−	12	−	−	12
Lei et al. (14)	2	49	None	hydatidiform mole	LC	VATS	1	NA	Thoracoscopic lower left lobectomy with mediastinal lymphadenectomy	−	−	−	3	−	−	3
Urabe et al. (19)	3	38	Poor physical condition	term pregnancy	NA	VATS	Multiple	80.1	Thoracoscopic right segmentectomy	−	6 cycles of EMA-CO	+	6	+	Surgical resection of pulmonary lesions	9
Li et al. (20)	4	31	None	Subclinical miscarriage	NA	VATS	1	168.1	Thoracoscopic left upper lobe segmentectomy	−	−	3 cycles of EP-EMA	13	−	−	13
Lewin et al. (21)	5	38	None	term pregnancy	Large cell LC	VATS	1	400	Thoracoscopic wedge resection and lobectomy	−	−	−	90	−	/	90
	6	49	vaginal bleeding	Miscarriage	LCA	PTNB, TS	1	2,204	Right lower lobectomy with mediastinal lymph node dissection	+	3.5 cycles of EMA/EP	−	45	−	/	45
	7	34	Irregular menses	term pregnancy	NA	TS	1	426	Right upper lobe segmentectomy	+	4 cycles of MTX	3 cycles of EMA/EP	22	−	/	22
Ahn et al. (22)	8	26	Delayed and relatively heavy menstruation	Suspected subclinical miscarriage	LC	PTNB, VATS	1	NA	Thoracoscopic right lower lobectomy with mediastinal lymph node dissection	−	−	6 cycles of EMA/CO	9	−	/	9
Abrão et al. (23)	9	31	Irregular vaginal bleeding	term pregnancy	Miscarriage, NSCLC	VATS	1	700	Thoracoscopic right lower lobectomy and systematic mediastinal lymphadenectomy	−	−	−	12	−	/	12
Sobecki-Rausch et al. (24)	10	20	None	term pregnancy	Ectopic pregnancy	VATS	1	50	Thoracoscopic wedge resection of the right	+	−	3 cycles of TP/TE	47	−	/	47

MTX, methotrexate; LC, lung cancer/lung carcinoma; VATS, video-assisted thoracoscopic surgery; EMA, etoposide, methotrexate and actinomycin D; CO, cyclophosphamide, vincristine; TS, thoracic surgery; PTNB, percutaneous transthoracic needle biopsy; NSCLC, non-small cell lung cancer; EP, etoposide, cisplatin; TP, paclitaxel, cisplatin; TE, docetaxel, epirubicin; +, positive; −, negative; NA, not available; /, blank.

Our patient presented with isolated pulmonary ETT lesions without uterine involvement and strongly required for preserving fertility. The personal management plan for this ETT patient had been fully discussed by the multi-disciplinary teams, including obstetrics and gynecology, thoracic surgery, and pathology. Following surgical intervention, a rigorous surveillance protocol is mandatory. Serum *β*-hCG levels should be monitored weekly until four consecutive negative results are achieved. Subsequently, the frequency is reduced to monthly for three additional negative cycles, followed by semi-annual monitoring indefinitely. For radiological surveillance, chest CT is recommended every 6 months; after 2 consecutive negative scans, the interval may be extended to annually. Beyond the fifth postoperative year, chest CT should be performed biannually for the remainder of the patient’s life. Additionally, annual TVUS is advised. During the follow-up period, if imaging remains unremarkable despite persistently elevated serum *β*-hCG, further diagnostic evaluation—including hysteroscopy and endometrial biopsy—is warranted to exclude ETT recurrence or metastasis and to determine the feasibility of continuing conservative management.

Unlike gestational CCA, ETT exhibits relative chemoresistance, making surgical resection the primary therapeutic mainstay. The FIGO stage and specific risk factors largely dictate the decision to initiate chemotherapy. For Stage I ETT, adjuvant chemotherapy is not universally mandated; however, it is strongly considered for patients with high-risk features, particularly a long interval (years) since the antecedent pregnancy or an elevated mitotic index. Conversely, Stages II–IV ETT necessitate a multimodal approach, combining cytoreductive surgery with intensive systemic chemotherapy to address occult or macroscopic metastatic disease (18). Given the biological similarities between ETT and PSTT, platinum-based multi-agent regimens are preferred over standard methotrexate-based protocols. The EP-EMA regimen (etoposide and cisplatin alternating with etoposide, methotrexate, and actinomycin D) remains the most widely adopted frontline therapy. For patients demonstrating resistance to EP-EMA or those with advanced systemic involvement, platinum-taxane combinations, such as TP/TE (paclitaxel/cisplatin alternating with paclitaxel/etoposide), serve as effective salvage or intensified alternatives. Among the 10 previously documented cases (Table 2), seven patients underwent pulmonary resection combined with adjuvant chemotherapy, all of whom remained recurrence-free throughout the follow-up period. These findings underscore that definitive surgical clearance remains the mainstay of ETT therapy, with systemic chemotherapy serving as a personalized adjunct based on disease staging and risk stratification.

This case highlights the clinical rarity of isolated pulmonary ETT. In women of reproductive age presenting with an isolated pulmonary nodule and elevated serum *β*-hCG, the lesion may be easily misdiagnosed as a primary lung malignancy or metastatic squamous cell carcinoma. Our study indicates that immunohistochemistry for GATA-3 and p63, combined with detailed gynecologic evaluation and pregnancy history screening, is critical for an accurate diagnosis. With a 4-year recurrence-free follow-up, we demonstrate that a lung-preserving resection without hysterectomy is feasible in patients with isolated pulmonary ETT and no uterine involvement. For young patients with a strong desire to preserve fertility, this approach, coupled with rigorous postoperative monitoring, achieves a favorable balance between oncologic safety and reproductive function preservation.

## Limitations

Despite the favorable outcome, this study has several limitations. First, as a single case report, our findings regarding the efficacy of fertility-sparing surgery lack broad generalizability to all patients with pulmonary ETT. Second, because a hysterectomy was not performed, the diagnosis of an “isolated” pulmonary lesion relies on the absence of clinical and imaging evidence (including TVUS, hysteroscopy, and endometrial biopsy) rather than a definitive uterine pathologic examination. Consequently, the presence of a regressed or microscopic primary uterine lesion cannot be entirely ruled out.

## Conclusion

The preoperative diagnosis of isolated pulmonary ETT is challenging due to the absence of distinctive clinical symptoms and imaging findings. In future clinical practice, when female patients, particularly those of reproductive age, demonstrate persistent mild elevations in serum *β*-hCG levels alongside pulmonary imaging revealing lesions, and the uterus shows no significant pathology, there should be a high index of suspicion for pulmonary ETT. Management of isolated pulmonary ETT can be customized on a case-by-case basis, and for patients desiring to maintain their fertility, resection of the isolated pulmonary lesion without hysterectomy remains a viable option.

## Data Availability

The original contributions presented in the study are included in the article/supplementary material, further inquiries can be directed to the corresponding author.
